# Bimekizumab and progressive pulmonary fibrosis in severe psoriasis: a case report

**DOI:** 10.3389/fmed.2026.1935395

**Published:** 2026-09-11

**Authors:** Alberto Corrà, Elena Biancamaria Mariotti, Valentina Ruffo di Calabria, Alessandro Magnatta, Luca Sanna, Virginia Corti, Marta Donati, Irene Bonanni, Rachel Daher, Lavinia Quintarelli, Elisabetta Rosi, Alice Verdelli, Marzia Caproni

**Affiliations:** 1 Rare Dermatological Diseases Unit, Section of Dermatology, Department of Health Sciences, USL Toscana Centro (ERN-SKIN), University of Florence, Florence, Italy; 2 Respiratory Medicine Unit, Careggi University Hospital, University of Florence, Florence, Italy

**Keywords:** bimekizumab, IL-17, interstitial lung disease, psoriasis, psoriasis treatment

## Abstract

Psoriasis is a chronic immune-mediated inflammatory disease frequently associated with systemic comorbidities, including an increased prevalence of interstitial lung diseases (ILDs). Progressive fibrosing ILDs are characterized by declining respiratory function and high morbidity, and emerging evidence suggests that type-17 immune pathways may contribute to both psoriatic inflammation and pulmonary fibrosis. Bimekizumab, a monoclonal antibody selectively targeting IL-17A and IL-17F, has demonstrated high efficacy in psoriasis, although its potential impact on fibrotic lung disease remains unclear. We report the case of a 58-year-old man with severe plaque psoriasis and concomitant desquamative interstitial pneumonia (DIP) with features of progressive pulmonary fibrosis. The patient presented with severe restrictive lung disease, reduced diffusing capacity, and exertional desaturation requiring oxygen supplementation. Previous treatments included topical therapies, acitretin, and intermittent systemic corticosteroids, which contributed to worsening skin disease. Bimekizumab was initiated after appropriate screening, resulting in rapid and complete skin clearance by week 12, with no adverse events. Over a 14-month follow-up, a progressive improvement in respiratory function was observed, including increased spirometric parameters, improved diffusing capacity, and enhanced exercise tolerance, with resolution of exertional desaturation and discontinuation of oxygen support during mild activity. No other therapeutic changes were made during this period. Given the role of IL-17 cytokines in inflammation and fibrosis, this observation suggests a potential effect of IL-17A/F inhibition on fibrotic lung processes. This case highlights a possible dual benefit of bimekizumab in patients with psoriasis and concomitant ILD and supports further investigation into shared pathogenic pathways and targeted therapeutic strategies.

## 1 Introduction

Psoriasis is one of the most common inflammatory dermatoses, affecting approximately 1%–5% of the global population. It is a chronic, relapsing condition that significantly impairs quality of life and is frequently associated with multiple comorbidities, including hypertension, obesity, type 2 diabetes mellitus, and dyslipidemia. Beyond these associations, psoriasis itself independently increases cardiovascular risk, likely due to persistent systemic inflammation related to disease severity and duration (1).

The pathogenesis of psoriasis involves a complex interplay between innate and adaptive immune responses, characterized by a self-amplifying inflammatory loop. Among the key pathways implicated, the IL-23/Th17 axis plays a central role, with IL-17 acting as a major effector cytokine (2). These immune mechanisms are not confined to the skin but reflect a systemic inflammatory process that may contribute to extra-cutaneous manifestations.

In this context, several studies have reported an increased prevalence of chronic pulmonary diseases among patients with psoriasis compared to the general population (3–6) Interstitial lung diseases (ILDs), in particular, may evolve into a progressive fibrosing phenotype characterized by worsening respiratory function, declining quality of life, and high mortality. Despite heterogeneous etiologies, these conditions share clinical and biological features, suggesting the involvement of common pathogenic pathways.

Patients affected by interstitial lung diseases (ILDs) may develop a progressive fibrosing phenotype characterized by increasing fibrotic changes on high-resolution computed tomography, worsening respiratory function, declining quality of life, and high mortality. Increasing evidence points to a central role of immune dysregulation, particularly involving T-cell–mediated responses.

In this context, type-17 immunity has emerged as a key contributor to fibrotic processes. T-cell dysfunction promotes the production of IL-17A and TGF-*β* in idiopathic pulmonary fibrosis, while elevated levels of IL-17A and IL-1β have been detected in bronchoalveolar lavage fluid. Similarly, upregulation of the IL-17 signaling pathway has been described in dermatomyositis-associated ILD and other fibrotic lung diseases, including those related to anti–aminoacyl tRNA synthetase antibodies. These findings suggest a potential mechanistic link between IL-17–driven inflammation and pulmonary fibrosis (7).

Bimekizumab is a humanized monoclonal antibody that selectively inhibits both IL-17A and IL-17F, approved for the treatment of moderate-to-severe plaque psoriasis, psoriatic arthritis, axial spondylarthritis and hidradenitis suppurativa. In the treatment of psoriasis, it has demonstrated superior efficacy in achieving high levels of skin clearance compared with selective IL-17A inhibition (8). However, the broader systemic effects of dual IL-17 blockade, particularly in fibrotic diseases, remain poorly defined (9).

Here, we report the case of a patient with severe psoriasis and concomitant ILD in whom treatment with bimekizumab resulted not only in complete skin clearance but also in a marked and unexpected improvement in respiratory parameters.

## 2 Case presentation

A 58-year-old man presented with a long history of chronic plaque psoriasis, for which he has undergone treatment with topical therapy either as monotherapy or in combination with systemic acitretin which was administered for 8 months before quitting for inefficacy. The patient was a former heavy smoker, having quit two years prior. At the time of first consultation, he was found to be affected by arterial hypertension and desquamative interstitial pneumonia, a subtype of idiopathic ILD, diagnosed with thorax computed tomography (CT) and transbronchial cryobiopsy. No familiar history of psoriasis or ILD were reported. Recent spirometries dating back to 19 and 7 months before respectively (Figure 1, T0 and T12) showed severe restrictive lung disease with reduced total lung capacity (TLC), reduced forced expiratory volume in 1 s (FEV1), reduced forced vital capacity (FVC) and a severe reduction of the diffusing capacity of the lungs for carbon monoxide (DLCO). A 6-minute walk test resulted in significant desaturation on exertion with minimal reported saturation of 83% and a gait interruption. Based on these findings, oxygen therapy was prescribed during physical activity, and the initiation of nintedanib was planned due to the presence of fibrotic changes on chest CT, characterized by interlobular septal thickening and traction bronchiectasis.

**Figure 1:**
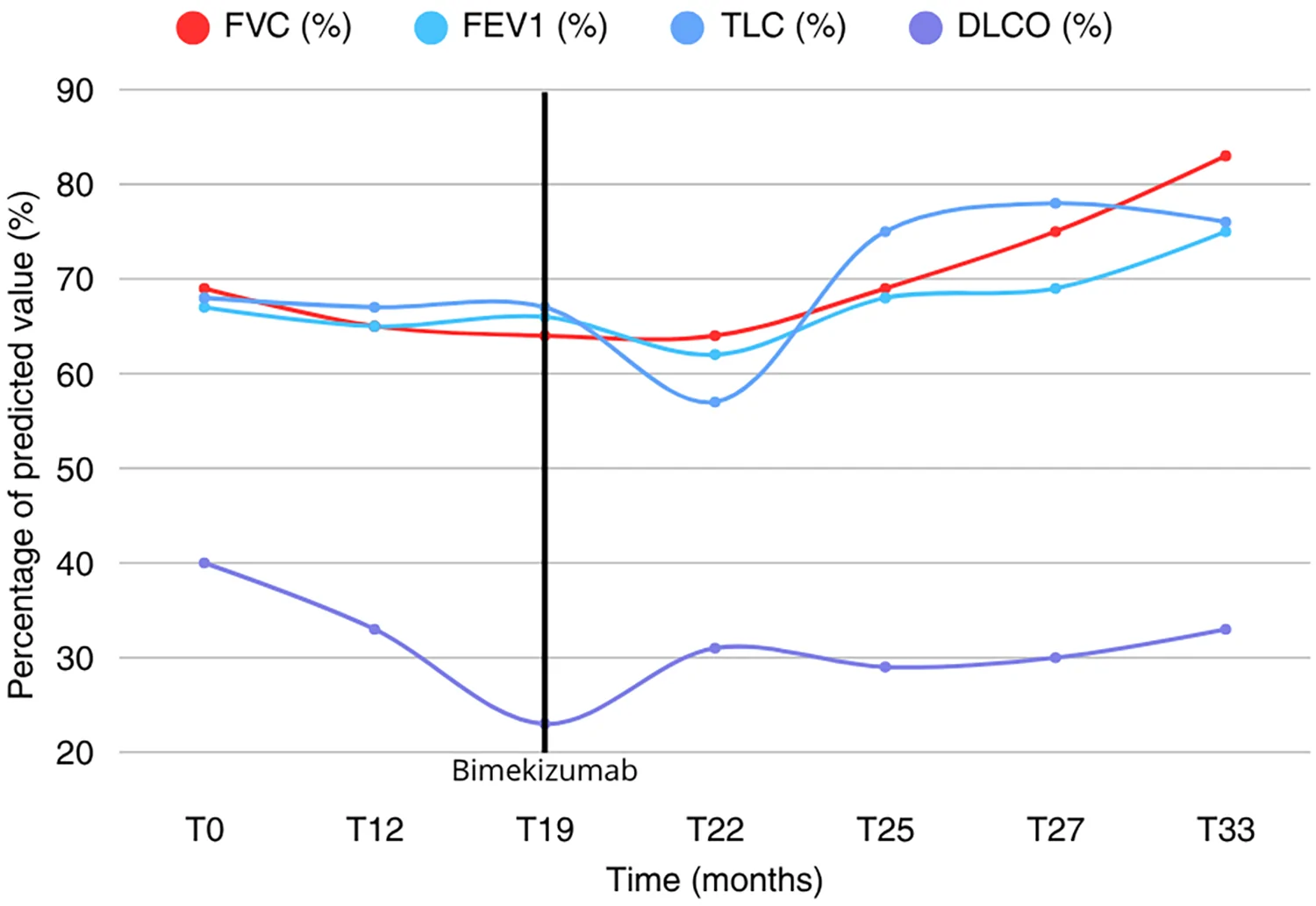
Longitudinal assessment of pulmonary function. FVC, FEV1, TLC, and DLCO are expressed as percentages of their respective predicted values at baseline (T0) and during follow-up through month 33 (T33).

To control the recurrent episodes of dyspnea and bronchitis he was repeatedly prescribed with short courses of systemic corticosteroids, which resulted in the secondary effect of worsening psoriasis and increasing the affected body surface area with PASI 41.9 at the time of first presentation.

Bimekizumab was initiated for the treatment of psoriasis, after screening for HIV, HBV, HCV and latent tuberculosis (Figure 1, T19) at a dose of 320 mg at weeks 0, 4, 8, 12, and 16, followed by administration every 8 weeks, according to the approved dosing regimen for psoriasis.

Rapid and sustained response with PASI100 was reached by week 12 in complete resolution of psoriasis plaques and no adverse effect reported (Figure 2).

**Figure 2:**
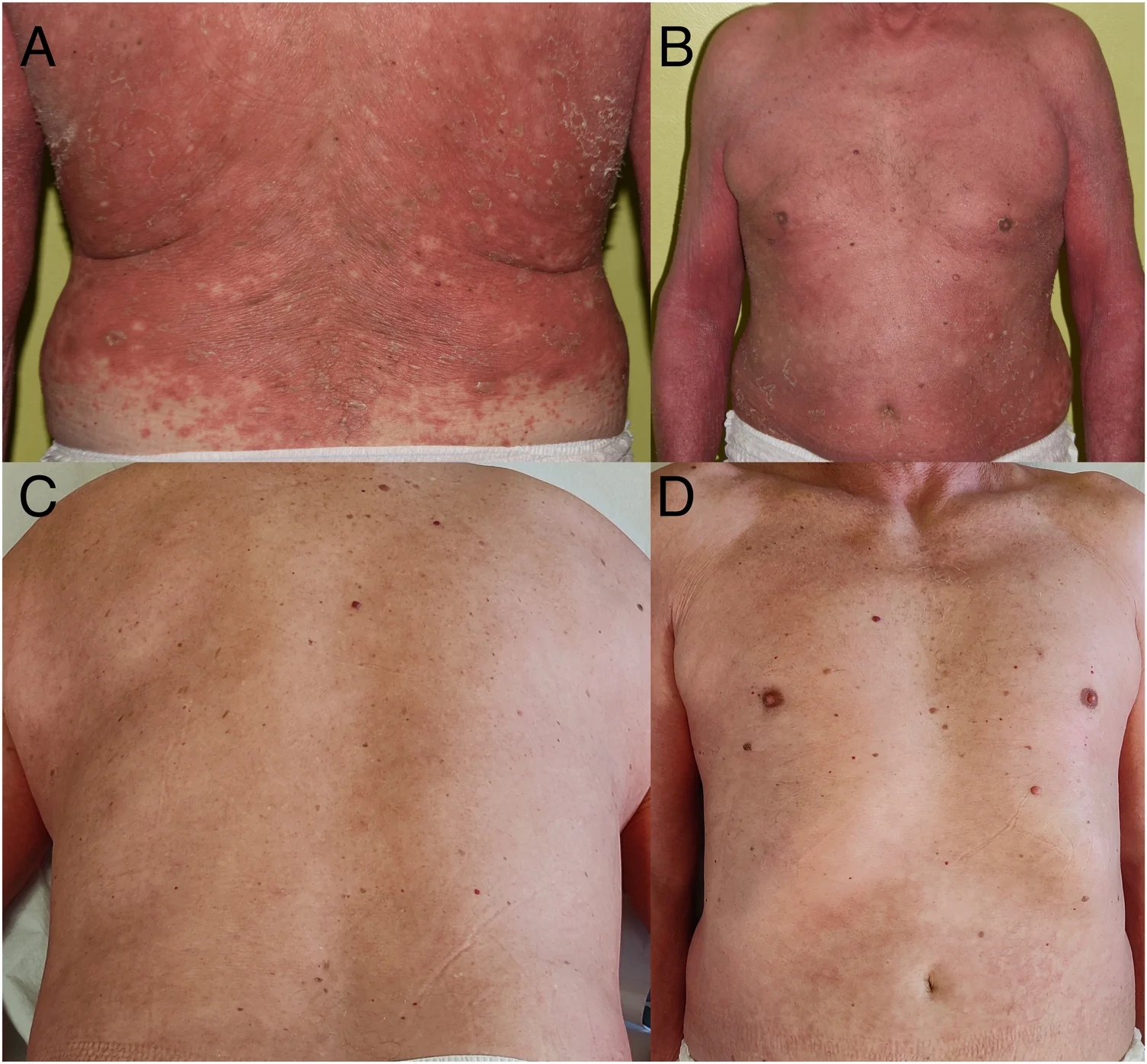
**(A,B)** clinical picture of the patients at the time of first presentation, showing plaque psoriasis with wide involvement of the trunk. **(C,D)** clinical picture 12 weeks after start of bimekizumab treatment, showing complete remission.

The treatment was then maintained while the pneumological follow-up showed a surprising 14-month trend of slow amelioration of respiratory parameters, as showed in Figure 1 (T22, T25, T27, T33).

Beyond the improvements observed in spirometry testing, the patient reported an even greater perception of improvement of his respiratory status, according to the 6-minute walking test performed 12 months later which did not report a significant desaturation on exertion (93%) allowing him to refrain from using oxygen support for mild physical effort. In view of this favorable clinical and functional evolution, along with radiological stability, nintedanib was ultimately not started (Figure 3).

**Figure 3:**
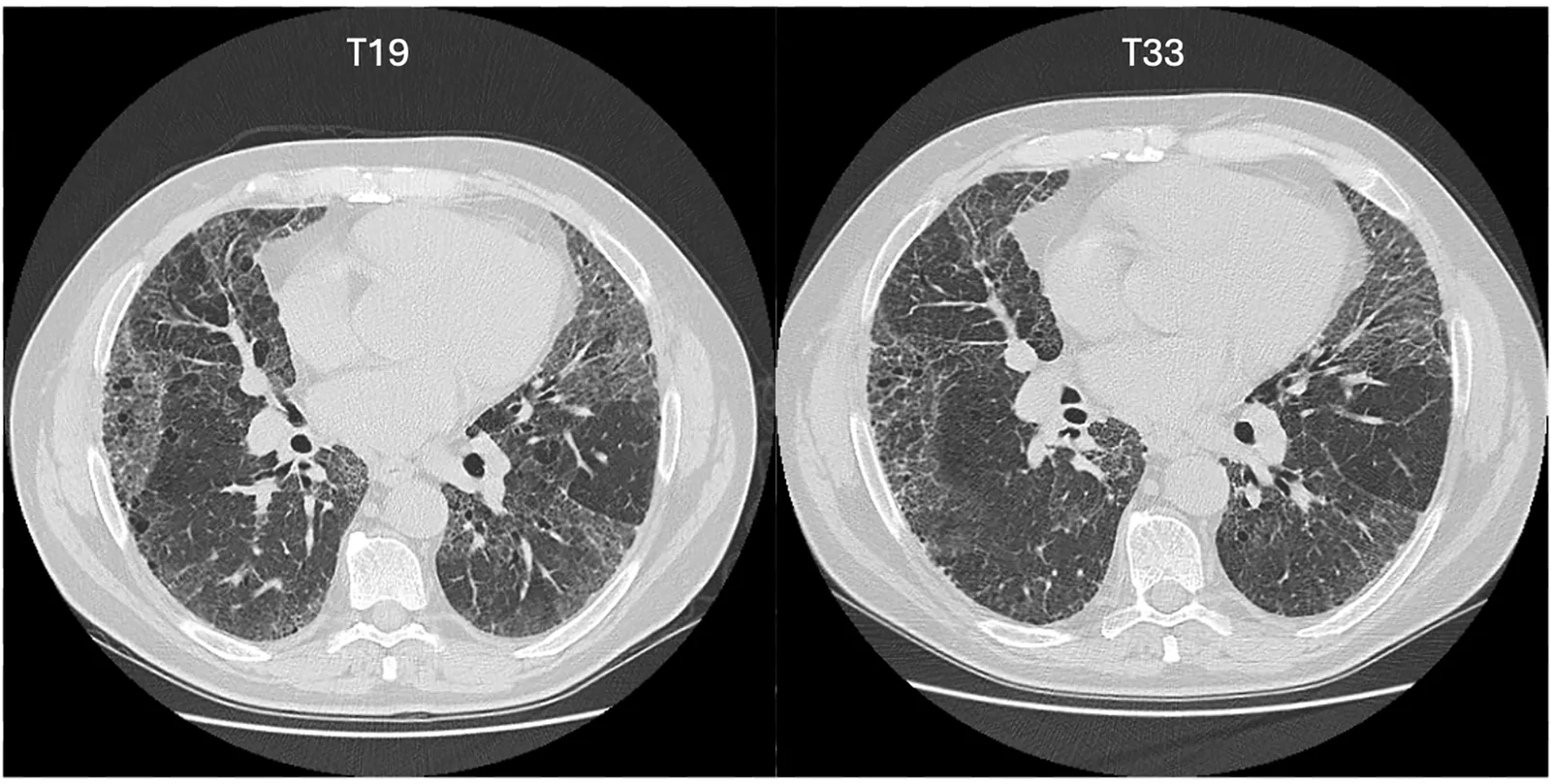
HRCT performed at timepoints T19 and T33 showing no substantial radiological progression of the pre-existing interlobular septal thickening and traction bronchiectasis.

## Discussion

Desquamative interstitial pneumonia is a rare form of ILD, primarily associated with smoking. It is characterized by the accumulation of macrophages in the alveolar spaces, leading to progressive respiratory impairment. Towards advanced stage disease, a fibrotic process can develop, thickening the alveolar septa and further worsening the respiratory parameters (10–12). This process is called progressive pulmonary fibrosis (PPF) and is defined by an interstitial lung disease showing the worsening of at least two among clinical symptoms, radiological findings and respiratory parameters in one year (13). Our patient met the criteria, considering the clinical respiratory worsening which led to oxygen supplementation, along with an absolute decline in FVC of ⩾ 5% and the decline in DLCO of ⩾10% within 12 months before presenting to our clinic for the treatment of psoriasis.

The unexpected progressive improvement in pulmonary function tests may be linked to the start of bimekizumab treatment. In support of this, a temporal coincidence appears considerable, as no other therapy changes were made in the same period. The course of PPF typically shows slow worsening, leading to progressive deterioration of respiratory parameters, while in this case the role of a reverting factor impacted on their evolution. Moreover, apart from the well-known IL-17 cytokines family activities in promoting pathogenic skin inflammation in psoriasis, increasing evidence support the role of IL-17 in fibrotic diseases. Indeed, IL-17 demonstrated a central role in fibrosis sustaining chronic inflammation and promoting the activation of stromal cells and fibroblasts in several organs. It contributes to tissue remodeling through the induction of epithelial-to-mesenchymal transition (EMT) and enhanced collagen deposition. Experimental murine and human models showed that inhibition of IL-17 signaling may mitigate liver fibrotic progression (13, 14) while elevated IL-17 expression has been observed in fibrotic processes affecting the myocardium (15) and the salivary glands affected by Sjogren's syndrome (16).

Furthermore, IL-17A and IL-17F appear to be significantly increased in several types of lesions in hidradenitis suppurativa, such as draining tunnels, inflammatory nodules, and non-draining tunnels. In two phase III trials, bimekizumab appeared to not only prevent tunnel progression but also to actively reduce the draining tunnel counts (17–19).

Regarding lung fibrosis, IL-17 has been implicated in the pathogenesis of idiopathic pulmonary fibrosis (IPF) (20). Experimental models using bleomycin-induced lung injury have shown that IL-17 can have both profibrotic and immunomodulatory effects, depending on the cellular context and regulatory signals. In fact, some studies report that IL-17 promotes neutrophilic inflammation and collagen production (21, 22).

Based on current evidence, it is reasonable to hypothesize that the patient described could be affected by PPF with strong IL-17-based pathogenetic imprinting which may have particularly benefited from a broad-spectrum anti-IL-17 therapy. Improvement in clinical and functional parameters support the possible effect of bimekizumab on ILD, since no other treatments were contemporarily started or adjusted for psoriasis or lungs. The smoking cessation may represent a confounding factor, since its contribution cannot be completely separated from the possible effect of bimekizumab. Nevertheless, the patient continued to show pulmonary impairment and progressive fibrotic changes despite stopping smoking approximately two years earlier. The subsequent improvement occurred after the initiation of bimekizumab. Even if the temporal association appears clinically significant, it does not establish certain causality.

Besides an unexpected clinical observation should not be interpreted as evidence supporting the use of bimekizumab as an antifibrotic treatment, our experience may inspire insights about the treatment of psoriasis in patients with concomitant ILD: if available, a treatment working on both diseases should be preferred to reduce the pharmacological load for the patient. However, it is likely that only a fraction of patients with ILD may benefit from IL-17 blockade. More studies about the pathogenetic relevance of IL-17 in ILD or PPF are obviously needed and the development of biomarkers to identify suitable candidates would represent a major advance toward personalized treatment of psoriasis.

## Data Availability

The original contributions presented in the study are included in the article/Supplementary Material, further inquiries can be directed to the corresponding author.
